# ‘Community evolution’ – laboratory strains and pedigrees in the age of genomics

**DOI:** 10.1099/mic.0.000869

**Published:** 2020-01-20

**Authors:** Matthew J. Dorman, Nicholas R. Thomson

**Affiliations:** ^1^​ Wellcome Sanger Institute, Wellcome Genome Campus, Hinxton, Cambridgeshire, CB10 1SA, UK; ^2^​ London School of Hygiene and Tropical Medicine, Keppel St, Bloomsbury, London WC1E 7HT, UK

## Abstract

Molecular microbiologists depend heavily on laboratory strains of bacteria, which are ubiquitous across the community of research groups working on a common organism. However, this presumes that strains present in different laboratories are in fact identical. Work on a culture of *Vibrio cholerae* preserved from 1916 provoked us to consider recent studies, which have used both classical genetics and next-generation sequencing to study the heterogeneity of laboratory strains. Here, we review and discuss mutations and phenotypic variation in supposedlyisogenic reference strains of *V. cholerae* and *Escherichia coli *, and we propose that by virtue of the dissemination of laboratory strains across the world, a large ‘community evolution’ experiment is currently ongoing.

Much of our current knowledge of bacteriology has been founded on the study of laboratory strains that are members of a species of interest. These strains are often deeply characterized, have had their genomes sequenced to completion, and are present in laboratories around the world. Standardizing on a handful of laboratory strains for molecular studies, rather than using environmental or clinical isolates, enables reproducible studies of a single strain’s genetics and physiology. This facilitates the stepwise accumulation of knowledge in research – as one group publishes on the physiology of a laboratory strain, another group can build upon that knowledge when they study their own stock of that strain. A large caveat to focusing on limited numbers of lab strains is that just as type strains define bacterial species taxonomically [1], a type strain is not the archetype of a species [2]. Similarly, it can be argued that we know more about our laboratory strains of bacteria at a genetic and biochemical level than we do about a species as a whole [3].

In a recent study, we and our collaborators revived a lyophilized stock of NCTC 30, a *Vibrio cholerae* first isolated in 1916 [4]. We sequenced the genome of this isolate, as well as examining its morphology and phenotype. Although we did not find many previous studies that had examined NCTC 30 [5–7] (Fig. 1), we took comfort in the fact that the cultures with which we worked behaved similarly to the findings reported in previous papers. We observed that NCTC 30 was phylogenetically distinct from pandemic *V. cholerae *, in agreement with a previous taxonomic study [6]. Concordant with another study, we found that NCTC 30 was less susceptible to ampicillin than NCTC 5395, another *V. cholerae* [7], and we identified a functional β-lactamase gene in the genome of NCTC 30 that we believe confers this phenotype [4]. All of this reassured us that although we were working with a bacterial stock that has been maintained for over 102 years, we were likely to be handling a descendent or close relative of the cultures studied in the past.

**Fig. 1.**
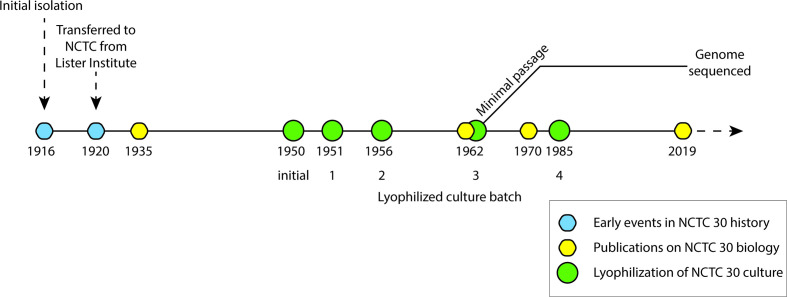
Overview of the curation of NCTC 30. NCTC 30 was isolated in 1916, transferred to the Lister Institute, and subsequently transferred into the NCTC collection in 1920. The strain was lyophilized in 1950 and four consecutive lyophilized stock batches have been prepared since then. Assorted manuscripts have studied NCTC 30 since its isolation, including [4–7]. Figure drawn using records in the supplementary data of [4].

However, a striking difference between our results and previous reports was the finding that our stock of NCTC 30 did not express flagella when examined microscopically, and was not motile on soft agar plates [4]. This disagreed with a previous report, which had described NCTC 30 as being flagellated [7]. Moreover, we were able to identify a mutation in our NCTC 30 genome, which we believe is responsible for this phenotype. Since it is wholly possible that spontaneous mutation due to long-term culture led to the loss of flagella in NCTC 30, we confirmed that this mutation is present in the batches of NCTC 30 currently available to purchase from Public Health England Culture Collections. Thus, researchers that now choose to study NCTC 30 ought to be working with a culture with the same genotype and phenotypes as our stock.

Discrepancies between what one ‘expects’ of a laboratory strain and how it actually behaves *in vitro* are a fascinating, and yet potentially problematic, aspect of bacteriology. Our study of NCTC 30 meant that we felt it timely to consider the variability of laboratory strains between and within laboratories, and the potentially fascinating community evolution that has been ongoing across these laboratories. Below, we address some recent examples of discrepancies between supposedly isogenic stocks of laboratory strains of bacteria, and between those strains and their reference genome sequence.

*V. cholerae* researchers typically make use of a small number of reference laboratory strains, which have been shared amongst laboratories worldwide and are used for genetic experiments, phenotypic comparisons between *V. cholerae* biotypes, etc. The initial *V. cholerae* reference genome sequence was determined in 2000 by Heidelberg and colleagues by sequencing the serogroup O1 biotype El Tor strain N16961 [8]. However, differences between a published genome sequence for a laboratory strain and the actual genome of the organism held in a laboratory stock can confound research findings. For instance, Val *et al*. used N16961 as a reference strain for studying the timing of *V. cholerae* chromosomal replication, but found discrepancies between the N16961 reference sequence and their results [9]. In their data, they identified an inversion flanked by rRNA operons in the genome of their N16961 stock relative to that of the reference sequence [9]. They confirmed that the chromosomal orientation in their N16961 cultures was common to N16961 stocks kept by other laboratories, as well as in other *V. cholerae* [9], and speculated that the inversion had occurred in the original stock of N16961 that was sequenced by Heidelberg *et al*. [8, 9].

Similar differences between published reference sequences and the genomes of laboratory strains have been identified in the case of the *V. cholerae* A1552 reference strain, the genome of which has been sequenced at least three times in the last 2 years [10–12]. Kemter *et al*., in a study of *Vibrio* chromosomal replication termination, used the N16961 reference sequence to design custom microarrays for this work, but found several inversions between their A1552 strain’s sequence and the reference N16961 genome [11]. The isolate sequenced by Kemter *et al*. contained an inversion characteristic of A1552, which was validated using long-read sequencing [11]. The genome sequence was published alongside the paper, and the sequenced strain was deposited in the DSMZ culture collection. Just as we have discussed for NCTC 30, purchase of A1552 from DSMZ ought to provide a researcher with the same bacteria as those characterized previously [11].

Discrepancies between expected and observed phenotypes can also confound genetic experiments in laboratory strains. An example of this was published in 2016, in which seven laboratory stocks of the *V. cholerae* reference strain C6706 were compared for their ability to be naturally transformed [13]. The natural competence of *V. cholerae* can be exploited by researchers to mutagenise *V. cholerae* experimentally [14–16]. Natural competence is induced in *V. cholerae* in response to being grown on chitin [14], and is also regulated by quorum sensing [17–19]. The master regulator of quorum sensing in *V. cholerae *, HapR, positively regulates genes, which encode components of the DNA-uptake apparatus, and negatively regulates expression of the *dns* nuclease gene [17, 20] (summarized in Fig. 2). It is well known in the *V. cholerae* community, for instance, that N16961 cannot be transformed naturally [14], due to a frameshift in the *hapR* gene [21, 22]. In the absence of HapR, *dns* is not repressed under conditions which would otherwise promote natural competence, leading to the degradation of exogenous DNA [20]. *hapR* mutations have been suggested to occur frequently in *V. cholerae* [22], and this has also been suggested to be as a consequence of the intrinsic bias towards collecting virulent isolates [15]; HapR downregulates the expression of cholera toxin and the toxin co-regulated pilus, principal *V. cholerae* virulence determinants (Fig. 2), and thus an isolate lacking functional *hapR* might cause a more acute disease, leading to hospitalization and the isolation of *V. cholerae* more likely to be added to a strain collection [15].

**Fig. 2.**
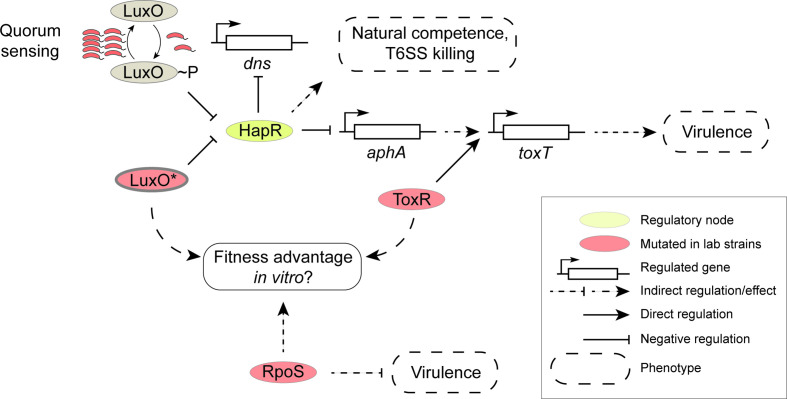
Laboratory strains of bacteria harbour mutations in several pathways which converge on increasing fitness *in vitro*. Phosphorylated LuxO (LuxO~P) decreases in concentration in *V. cholerae* cells as the population of bacteria grows, measured by quorum sensing. LuxO~P represses HapR; thus, at high-cell density, HapR abundance increases as the concentration of active LuxO~P decreases. HapR influences quorum-dependent phenotypes such as natural competence and virulence, the latter by repressing *aphA*, an activator of virulence gene expression in *V. cholerae* [26]. Mutations in *hapR* and *luxO* therefore influence these phenotypes. Other mutations can prevent the activation of the virulence regulon, such as in *toxR*, or producing constitutively activated LuxO* mimicking low-cell density quorum sensing, thereby forcing the maintenance of HapR repression of the virulence regulon. Similarly, mutations in *rpoS* are reported to repress virulence, and therefore increase the fitness *in vitro* of these mutants relative to wild-type cells.

Various researchers reported that they were unable to effect natural transformation in C6706, leading to Stutzmann and Blokesch’s study [13]. They found that the seven C6706 stocks examined had wild-type *hapR* alleles, but four contained a mutant *luxO* allele predicted to encode a mutant LuxO^G333S^ protein [13]. This correlated with impaired quorum sensing-dependent phenotypes, such as chitin-induced transformation and reduced type VI secretion system-mediated killing of *E. coli* prey bacteria, as well as reduced levels of *hapR* transcription and a concomitant change in the expression of the HapR regulon [13]. They concluded that the circulation of quorum sensing-deficient strains of C6706 necessitated that researchers working with this strain should confirm the *luxO* genotype of their laboratory stocks, if discordant natural transformation phenotypes manifest. They suggested that the presence of the same *luxO* allele in these four stocks meant that the same *luxO* mutant strain had been passed amongst four laboratories [13].

It has been shown that prolonged maintenance of toxigenic *V. cholerae* on agar stabs can select for spontaneous nontoxigenic mutants, which arise due to mutation in the *toxR* promoter [23]. These *toxR* mutants do not express the ToxR regulon, including the cholera toxin, and gain a competitive growth advantage over toxigenic cells *in vitro* [23] (Fig. 2). Likewise, luxO* mutations producing constitutively active LuxO have been reported to arise in *Vibrio fischeri* cultures that are maintained in stationary phase for prolonged periods of time [24]. These luxO* mutants gained a fitness advantage over wild-type *V. fischeri* under the conditions used in the study [24]. The mutant LuxO^G333S^ protein found in C6706 stocks has been shown to mimic constitutively active LuxO [25] (Fig. 2). It is plausible that C6706 luxO* mutants possess a competitive advantage over their wild-type counterparts, leading to the isolation of such mutants during continuous passage and their subsequent dissemination to laboratories worldwide. It is also possible that these luxO* mutants express elevated levels of virulence determinants compared to the wild-type C6706 (Fig. 2). Mutations affecting the interactions between LuxO, HapR and AphA directly modulate virulence and cholera toxin expression in *V. cholerae* [26] (Fig. 2), and since C6706 is a lab strain commonly used for studying the regulation of *V. cholerae* virulence, mutants may inadvertently have been selected by researchers that express greater, more detectable, levels of virulence determinants, potentially distorting our view of the evolutionary trajectory of natural isolates.

*V. cholerae* are not the only bacteria susceptible to lab-to-lab variation in supposedly isogenic strains. Numerous examples of the same phenomenon in *E. coli* have been reported. Although the prototype *E. coli* K-12 was first isolated in 1922, from a convalescent diptheria patient in Palo Alto, CA, USA [27], its descendants continue to be used by researchers worldwide. In 2006, Hayashi *et al*. [28] reported genome sequences for two such laboratory strains, MG1655 and W3110, in a manuscript which accompanied the announcement of the Keio mutant collection [29]. The Keio mutants were constructed in BW25113 [30], a strain of *E. coli* derived from W1485, a common ancestor both of MG1655 [27, 31] and W3110 [27, 32]. The pedigree for BW25113 was reported in great detail upon publication of the Keio collection [29], and its genome sequence was subsequently determined [33]. Although an in-depth review of *E. coli* strain pedigrees is beyond the scope of this article, it would be remiss not to mention the work of organizations such as the *E. coli* Genetic Stock Center (CGSC) and Barbara Bachmann, its former curator, in maintaining extensive records of strain provenance, genetic description and pedigree [27, 32, 34]. Hayashi *et al*. reported heterogeneity amongst stocks of W3110 from laboratories across Japan, which they linked chiefly to IS element transposition events, which may have occurred during storage in stabs [28] – it is accepted that genome mutation takes place while *E. coli* is maintained as a stab culture [35]. They speculated that the limited variation that they observed between W3110 and MG1655, despite the two strains having been separated from one another over 50 years prior to their publication, might have been a consequence of using lyophilization as a storage method for these strains [28, 36].

Similarly, a recent study by Desroches *et al*. investigated the provenance and evolution of NCTC 86, the Escherich type strain of *E. coli* [37]. In this work, they sequenced cultures of NCTC 86 from stocks at NCTC, the ATCC, DSMZ and Centre de Ressources Biologiques de l’Institut Pasteur, as well as using two additional previously published sequences from NCTC stocks [38, 39] (a third sequence of this isolate was reported in [40] but not included in this analysis). The authors found striking heterogeneity in the genomes of these supposedly identical *E. coli* cultures, ranging from differences in plasmid replicon sequences being present in various stocks, to single-nucleotide polymorphisms and inactivating mutations in mutation-prevention pathways [37]. The authors were able to correlate some of these differences to the records of how NCTC 86 was shared between these culture collections, and from where the sequenced isolates were obtained [37]. A similar study was performed in *Campylobacter jejuni *, where 23 stocks of the laboratory strain NCTC 11168 from across the UK were both sequenced and phenotyped [41]. These *C. jejuni* stocks varied in their growth rates, motility, virulence, and the types and numbers of mutations in their genome, despite ostensibly being the same strain.

Just as certain pathways seem to be commonly mutated in *V. cholerae* laboratory strains (e.g. quorum-sensing pathways), there are mutations common to lab strains of other species. For instance, mutations in *rpoS* are found in the W3110 and NCTC 86 strains discussed above [28, 37]. *rpoS* mutations are known to occur during the process of laboratory adaptation [42, 43], or as a consequence of transferring bacterial strains between laboratories [44]. It is important to note, however, that the increased fitness of *toxR* mutant *V. cholerae* relative to wild-type cells is *rpoS*-independent [23]. Mutations in *rpoS* can confer a growth advantage to *E. coli* in a laboratory (e.g. grown at 37 ˚C in Luria–Bertani or minimal media) [45], and contribute to virulence attenuation associated with laboratory strains, such as in the LT2 strain of *Salmonella enterica* serovar Typhimurium [46] (Fig. 2). The gain of fitness conferred by these laboratory-acquired mutations confers a fitness advantage on these mutants relative to the wild-type, and appears to be a theme common to the discussion above. Whether mutations are acquired in quorum sensing [24], virulence regulons [23] or in *rpoS* [42–44], there appears to be a convergence on laboratory culture environments selecting for mutants that have a selective fitness advantage over the wild-type (Fig. 2), just as occurs in *in vitro* artificial evolution experiments [47, 48].

The discussion above illustrates the fact that phenotypic differences between laboratories’ strains offer an interesting opportunity to dissect the scientific basis underpinning those differences. This necessitates that researchers are willing to share their isolates with one another, or that they deposit important isolates in culture collections. The use of appropriate preservation and meticulous record curation means that a fellow researcher who orders a bacterial stock reported in a paper, from a culture collection, can be more confident that the organism they receive is that which produced the phenotypes in a previous publication [49]. Moreover, as the genome sequences of strain collections become freely available, the research community will benefit from the availability of biological and bioinformatic resources in which high levels of confidence can be had.

Simply by virtue of disseminating strains to different laboratories, researchers may have unwittingly conducted a natural evolution experiment. Comparing lab strains across different groups could present interesting opportunities to study evolution, or to resolve differences that are observed, such as explaining the presence of C6706 *luxO* mutants in laboratories’ strain collections. A fascinating exercise, for instance, might be to attempt to retrieve stocks of NCTC 30 from laboratories worldwide, which might contain their own stocks, and to perform a comparison both phenotypically and genomically of these stocks. If data on the provenance of each isolate was available and could be used to construct a pedigree of how and when NCTC 30 was disseminated to each laboratory, this could be compared to the phylogenetics of the isolate. This might allow for identifying when phenotypes changed and mutations were accumulated over the course of the strain’s propagation. Although *V. cholerae* NCTC 30 has inspired this example, and we have discussed *Vibrio cholerae* and *Escherichia coli* predominantly in this paper, precisely the same ‘community evolution’ experiment is likely to be ongoing in laboratories worldwide. Bachmann [27] lamented in 1996 that ‘the derivation of many strains being isolated today can never be traced because of the failure of some laboratories to maintain adequate records of strain constructions’. Now, with the ever-decreasing cost of bacterial genome sequencing, phylogenetic analysis of sequenced isolates with even partial records of their provenance may enable the recapitulation of pedigrees, and in the absence of records of strain exchange but with the genome sequences of the strains, perhaps to work out ‘who gave what to who’ using a phylogeny, just as in studies of bacterial transmission (e.g. [50]).
